# Automated abstraction of clinical parameters of multiple myeloma from real-world clinical notes using large language models

**DOI:** 10.1186/s12911-026-03345-z

**Published:** 2026-01-28

**Authors:** Alana O’Brien Del Campo, Dmytro Lituiev, Gowtham Varma, Mithun Manoharan, Sunil Kumar Ravi, Avinash Aman, Ankit Kansagra, Joel Greshock, AJ Venkatakrishnan, Ashita S. Batavia

**Affiliations:** 1 Johnson & Johnson Innovative Medicine, 301 Binney Street, Cambridge, MA 02142 USA; 2 Johnson & Johnson Innovative Medicine, 1 Johnson & Johnson Plaza, New Brunswick, NJ 08933 USA; 3 nference, 3rd, 4th & 5th Floor, Indiqube Golf View Homes, 3rd Cross, Rustam Bagh Layout, NAL Wind Tunnel Main Road, Murugeshpalya, Bengaluru, 560017 India; 4 Johnson & Johnson Innovative Medicine, 920 US Route 202 South, Raritan, NJ 08869 USA; 5 Johnson & Johnson Innovative Medicine, 965 Chesterbrook Boulevard, Wayne, PA 19087 USA; 6 nference, One Main Street, Suite 400 East Arcade, 4th Floor, Cambridge, MA 02142 USA

**Keywords:** Multiple myeloma, Real world data, Natural language processing, Large language models

## Abstract

**Background:**

Real-world evidence (RWE) is increasingly recognized as a valuable type of oncology research but extracting fit-for-purpose real-world data (RWD) from electronic health records (EHRs) remains challenging. Manual abstraction from free-text clinical documents, although the gold standard for information extraction, is resource-intensive. RWD generation using natural language processing (NLP) has been limited by performance ceilings and annotation requirements, which recent LLMs improve on. Multiple myeloma (MM) is the second most common hematological malignancy, with many opportunities for RWE to expand knowledge of disease and treatment. We evaluate new NLP workflows in abstracting MM-related clinical data fields from de-identified EHRs.

**Methods:**

NLP workflows (BERT and Llama-based, using various prompt types) were developed for 12 MM-specific data fields and evaluated with manually curated data from 125 clinical notes. Statistical analysis was conducted to evaluate characteristics of models and data associated with F_1_ scores. For 200 randomly selected patients, three illustrative data fields (MM status, transplant status, and extramedullary disease) were extracted with a corresponding timestamp from all patient notes within a 120-day window of the index MM diagnosis date using the best-performing Llama workflow. Abstracted data field labels were then plotted on a timeline to display the frequency with which these data fields are documented in patient records.

**Results:**

Average F_1_ across the 12 data fields for the best Llama and BERT workflows was 0.82 and 0.65 respectively. Best workflow performance ranged across the data fields (F_1_ = 0.59–0.99). Statistical analysis of the results showed model size, inter-rater reliability (IRR), variable type, and prompt design significantly predicted workflow performance, in descending order of significance (*p* < 0.05).

**Conclusion:**

Performance improvements with larger LLMs and chain-of-thought prompting was greater in data fields with greater difficulty of abstraction. IRR can prioritize NLP resources, increasing efficiency of RWD generation without sacrificing data quality. Strategic selection of NLP tool using the proposed framework has the potential to inform planning of RWD generation, ultimately accelerating insights from RWE.

**Supplementary Information:**

The online version contains supplementary material available at 10.1186/s12911-026-03345-z.

## Background

Multiple myeloma is the second most common hematological malignancy, with nearly 200,000 cases and 120,000 deaths estimated in 2022 [1]. In the past decade, multiple myeloma (MM) patients and providers have seen a rapid expansion of therapeutic options, creating greater need for real world evidence (RWE) that can expand on findings from randomized controlled trials [2–5]. Electronic Health Records (EHRs) and administrative claims contain a wealth of real-world clinical data capturing diagnoses, treatments and outcomes. Many useful data elements exist in the structured data, such as diagnosis codes, administered medications and procedures. However, key concepts for creating trial-like cohorts and assessing outcomes, such as transplant eligibility and certain International Myeloma Working Group (IMWG) criteria, are documented only in unstructured clinical notes. This data must be extracted and structured to create fit-for-purpose real-world data (RWD) usable for evidence generation, often in combination with structured data fields [6, 7]. 

Manual abstraction continues to be the gold standard for clinical information extraction (IE) from unstructured text. However, the time and resources required to manually abstract numerous clinical data fields may ultimately constrain RWE generation. Rules-based natural language processing (NLP) provides some automation in this process, although with substantial upfront investment of time. Examples of rules-based NLP in multiple myeloma have shown success in abstracting MM clinical parameters such as diagnosis, staging and select lab values, where these systems have achieved F_1_ scores above 0.9 [8–10]. Other parameters, such as MM subtype identification, have substantially lower performance with these approaches [10]. Examples of other machine learning applications in MM are more prevalent, such as developing digital pathology algorithms for differential cell counts in bone marrow aspirates and predictive models for ECOG score and outcome [11–13]. Applications such as these can and do use NLP abstraction to obtain ground truth labels or independent predictor variables from unstructured text [11].

In recent years, NLP techniques based on transformer language models have shown great potential for further automating this process [14]. Transformer models can be broadly categorized into encoders such as Bidirectional Encoder Representations from Transformers (BERT) typically used for discriminative tasks like classification and named entity recognition (NER), and decoders (such as GPT and Llama) and encoder-decoders typically used for tasks requiring text generation. Recent decoder models typically have at least 1 billion parameters compared to earlier encoder models (e.g., BERT with 300 million parameters). To our knowledge, use of these more recent NLP techniques in MM and application to a broader set of MM clinical parameters has not yet been conducted.

Until recently, NER-based IE using BERT models has been a popular NLP approach due to their strong performance across many tasks [15–17]. The effectiveness of BERT models for clinical IE has previously been demonstrated in tasks like identifying drug-related adverse events, extracting clinical symptoms and real-world disease outcomes [18–20]. Their success notwithstanding, BERT models pose certain limitations to investigators. BERT models, while highly parameterized compared to traditional NLP techniques, still have fewer parameters than most of the newer large language models (LLMs) and often require task-specific training on manually labeled datasets [21, 22]. Also, the narrower context window of most BERT models limits their ability to infer concepts that are typically presented over multiple sentences or paragraphs, such as transplant eligibility [23]. 

More recent generative LLMs have more parameters and larger context windows. When pre-trained on large corpora, these newer LLMs promise high off-the-shelf performance in clinical IE tasks without task-specific training, particularly for complex concepts [24]. These advances in LLMs thus hold promise to accelerate development of high quality real-world datasets by mitigating the need for extensive manual data curation and fine-tuning.

Early applications of generative LLMs for clinical IE predominantly use Flan and GPT family models, and demonstrate wide-ranging performance (accuracy and F1 statistics both ranging from low 40s to high 90s percentage), primarily due to three contributing factors [25–30]. First, complex data fields, such as adverse social determinants of health, challenge all NLP techniques including LLMs [31, 32]. Model size, which impacts pre-training capacity, drives off-the-shelf performance of LLMs. And finally, reasoning-based prompting techniques like chain-of-thought purport to help a generally pre-trained LLM handle specialized tasks better than zero-shot learning [33, 34]. 

Investigators planning RWD generation from unstructured text can find newer LLMs to be a powerful tool, but the lack of comparability between published studies hinders generalization of results to inform IE strategy. Off-the-shelf accuracy of LLMs is unpredictable, obfuscating the time and effort required to produce a reliable data field. Prompt engineering is an art more than a science, with few guardrails to guide users on improving performance. Although larger LLMs often achieve better results, they are more resource-intensive on multiple dimensions. Larger LLMs have higher token costs (e.g., Llama 3 70B tokens cost roughly five times as much as Llama 3 8B tokens). Furthermore, total cost of operation is increased by the hardware needed (the 70B model requires approximately 10-fold more GPU VRAM than 8B). In fact, running Llama 70B is infeasible with a single consumer-grade GPU, requiring more engineering time and expertise. The total cost of operating will be specific to the circumstances of the operator, but can be assumed to be substantially more for larger models.

Instead of defaulting to a single NLP technique for all data fields, efficient data generation balances accuracy versus time and resource demands [35]. Our objective in this research is quantify the impact of IE design choices and provide investigators with an approach to tailoring efficient LLM usage. Ultimately, we hope this will accelerate generation of high-quality MM RWD.

In this research, we utilize several NLP models (Llama 3 8B, Llama 3 70B, and BERT) in workflows to extract clinical information from the EHR notes of patients diagnosed with MM. The Llama family provided the best-performing open-source LLM at the time of analysis, based on the published benchmarks [36]. It provides a closely-related set of models that enables comparison of model size, while also being a novel contribution as no literature that we are aware of describes Llama model performance in real-world data curation. We evaluate NLP workflow performance against a manually abstracted reference dataset. We analyze the impact of text ambiguity, model size and chain-of-thought prompting on newer LLM performance. Finally, we use the best NLP workflow to extract three data fields for all recent notes in select patient charts, demonstrating enhanced information availability using the entirety of the observable patient journey.

## Methods

### Data source & selection

This study analyzed de-identified EHR data from a network of tertiary clinical centers tied to an academic medical center (AMC) in the United States through the nSights Platform from nference, a real-world data provider. nference, in collaboration with the AMC partner that provided the de-identified data for this study, has established a secure data environment, hosted by and within the AMC, that houses the AMC’s de-identified patient data. Data used in this analysis have been extracted from this environment using an established protocol for data extraction, aimed at preserving patient privacy. A full description of the de-identification model used can be found in Murugadoss et al. (2021) [37]. 

In accordance with the Code of Federal Regulations, 45 CFR 46.102(e) [1], analysis of de-identified data does not constitute human subjects research and therefore does not require IRB review. The data have been de-identified pursuant to an expert determination in accordance with the HIPAA Privacy Rule (45 CFR 164.514(b) [1]).

#### Patient population and selected notes

A cohort of MM patients was created and notes for IE were sampled from their charts. The study cohort (*n* = 3,793) included patients with MM diagnosis and treatment between January 1, 2019, to March 31, 2024. Patients with MM were identified using two occurrences of diagnosis codes of 203.0* (ICD-9), C90, and C90.0* (ICD-10). The first occurrence of MM diagnosis code was considered the diagnosis date. Patients were required to have at least one encounter recorded within six months of the diagnosis date and another encounter six months after the diagnosis date.

The notes database (*n* = 250 notes) was created by selecting one instance of provider-documented unstructured text associated with an encounter, pathology testing, or imaging study, from the charts of 250 patients randomly selected from the study cohort. Study cohort and note selection criteria are available in Supplementary Note 1. This notes database was randomly divided into development and test sets of 125 notes each.

### Clinical concepts and data fields

13 MM-related data fields were chosen. (See Table 1). All fields were text-based, provider-documented values (i.e., not derived from structured data fields such as ICD codes, medications, or timestamps). Subject matter expertise from a hematologist-oncologist was incorporated in selecting the 13 data fields to reflect the majority of key data that would be needed for clinically precise RWE generation. Data fields were reviewed for high annotation quality and agreement in pilot rounds to ensure high quality ground truth dataset.

**Table 1:** Data fields of interest contained in healthcare provider (HCP) notes

Concept	Data field	Value type and range
MM disease state	*1. MM diagnosis*	Categorical Yes / Likely / No / Unspecified*
	*2. MM status*	Categorical *Newly diagnosed / Relapsed / Refractory / Remission*
	*3. MM diagnosis date*	Datetime *YYYY-MM-DD*
Transplant eligibility & status	*4. Transplant eligibility & status*	Categorical Eligible / Eligible but deferred / Ineligible / Performed / Unspecified*
Eastern Cooperative Oncology Group (ECOG) performance status scale	*5. ECOG score*	Integer *0—5*
Plasmacytosis	*6. Plasmacytosis percentage*	Integer *0-100%*
MM-related bone lesions	*7. MM-related bone lesion presence*	Binary Yes / No / Unrelated / Unspecified*
Extra-medullary disease (EMD)	*8. EMD presence*	Binary Present / No / Unspecified*
	*9. EMD location*	Binary Paramedullary / Soft tissue / Unspecified*
HCP-documented first line therapy (FLT)	*10. FLT regimen*	Categorical *Set of drugs and procedures*, mapped to NDC or RxNorm and HCPCS or CPT**
	*11. FLT start date*	Datetime *YYYY-MM-DD*
HCP-documented FLT response	*12. FLT response*	Categorical *IMWG 2016 response categories: Stringent Complete Response / Complete Response / Very Good Partial Response /* *Partial Response / Minimal Response /* No Response / Progressive Disease / Unspecified*
	*13. FLT response date*	Datetime *Formatted as YYYY-MM-DD*

*Unspecified indicates no mention in text

**NDC: National Drug Code, RxNorm: National Library of Medicine (NLM) issued normalized naming system for generic and branded Drugs. HCPCS: Healthcare Common Procedure Coding System. CPT: Current Procedure Terminology

### Manual data curation

A reference dataset for the 13 data fields was created for prompt development and NLP workflow testing. The reference dataset was created by two independent abstractors with arbitration by a third abstractor.

Inter-rater reliability (IRR), measuring annotators’ agreement, was calculated to estimate the degree of abstraction difficulty for the data fields. IRR was assessed in two ways. First, Krippendorff’s α (K-α) was calculated. An overall average K-α of ≥ 0.8 for the test dataset was considered acceptable [38]. Secondly, the agreement between each abstractor and the final arbitrated label was evaluated using an average F_1_ score between each annotator’s and the consensus labels. This F_1_-based IRR (subsequently referred to as IRR-F_1_) was used in statistical analysis of the NLP workflows described below. Supplementary Note 2 contains abstraction protocol and metrics (K-α and F_1_ score for each data field, class distribution) and low-count class combinations.

### NLP-based methods for information extraction

Five LLM workflows for extracting information on the selected data fields were developed: four workflows utilizing Llama models and one workflow utilizing BERT.

#### Llama workflows

Meta’s Llama 3 family of models provided an open-source, privately deployable LLM with small and medium-sized model sizes [36]. The small model (8 billion parameters) has lower performance on all benchmarks assessed at release but is more computationally facile [36]. The medium model (70 billion parameters) offers advanced natural language capabilities, more promising for challenging clinical text, but required more computational infrastructure to run [36]. (A 405 billion parameter model was released after the start of research and thus not included.)

The small (8B) and medium (70B) models were run in private compute clusters to protect deidentified clinical text. Performance evaluations were done using a temperature of 0.1 and a top-K (the number of highest probability token options used for sampling) value of 1 for reproducible results. Sensitivity analysis was performance for additional top-K and temperature values.

Zero-shot-learning (ZSL) was selected for default performance and chain-of-thought (CoT) prompting was compared as a common reasoning-method technique. Bespoke prompts for each data field were systematically designed using the development set. Prompts were refined based on errors generated until achieving average abstractor F_1_ for the respective field or when subsequent modifications did not yield performance improvements. Final prompts are available in Supplementary Note 3. The model was prompted to return a JSON-structured response. The syntax of JSON output was parsed and standardized to pre-specified labels using post-processing logic.

During post-processing, the raw JSON emitted by the Llama workflows was enforced with a strict schema so that every field was parseable, on scale, and comparable against the ground truth labels. Briefly, it validated date strings, coerced categorical outputs to predefined bins, harmonized brand names to drug ingredients for FLT regimens, and rejected or flagged values that fall outside allowed ranges. When the JSON was malformed, structured repair was attempted up to three times, after which the field was marked “Unspecified” with a failure code. These steps ensured the LLM outputs conform to the target data dictionary before accuracy scoring.

These four Llama workflows (i.e., model-prompt combination) are referenced throughout using respective model size and prompting technique (e.g., 70B-CoT for Llama 3 70B with chain-of-thought prompting).

#### BERT workflow

A BERT workflow, earlier developed for a broad range of biomedical tasks on nference data, was deployed as the baseline technique. A pipeline of six proprietary BERT-based classification models includes: (1) named entity recognition model trained to detect 27 entity types; (2–4) qualifier models: subject, temporality and certainty models; (5) concept association model for “problem-location”, “problem-severity”, “lab data-value”; and (6) date association model for “variable-date” entity pairs. These proprietary models are fine-tuned versions of SciBERT cased [39] (basis for models 1–5 specified above) and ClinicalBERT [40] (basis for model 6). The base models underwent further supervised fine-tuning for IE tasks on annotated sentences from clinical document texts of the nference nSights database, but not specifically on MM patient note database.

For each data field, relevant synonyms derived from tokenization of the development set were curated and incorporated. The BERT pipeline output was refined using regular expression models and business rules developed using the development set. BERT workflow development approach, performance metrics, and rules are available in Supplementary Note 4.

### Statistical analysis

Macro-F_1_ scores were used to evaluate the performance of the NLP workflows. For multi-label fields such as dates, macro-F_1_ score was substituted with a weighted F_1_, calculated as defined in Supplementary Note 1. Spearman’s rho was calculated for numeric fields. Visualization and exploratory data analysis was conducted in Python (3.10.6) using pandas (2.2.2), numpy (1.26.4), scipy, matplotlib (3.9.1), seaborn (0.13.2), and plotly (5.23.0) (https://plot.ly/). [41–46].

Statistical analysis of LLM workflow performance for each data field included pairwise comparisons and, for the four Llama workflows, ANOVA with univariate regressions for binary variables or Tukey HSD post-hoc testing for categorical ones. Independent variables included in the ANOVA were: model size, prompt design style, data field type (numeric, binary, or categorical), and IRR-F_1_. IRR-F_1_ statistics describing combined development and test sets were used. Statistical analysis was performed in RStudio (2023.06.1), R (4.3.1) and visualizations were performed in ggplot2 (3.5.1) [47, 48]. 

### Evaluation of LLM-extracted events against structured diagnosis dates

For 200 randomly selected patients, the best-performing Llama workflow for three illustrative data fields (*MM type*, *transplant status*, and *extramedullary disease*) was deployed on all routine clinical notes within 120 days of MM diagnosis date as determined by ICD codes. All abstracted labels were plotted on a timeline using the timestamp of the note to visualize the timing, frequency, and distribution of the label occurrences around the structured diagnosis date.

Timestamp identification was derived in a two-step process. For each detected label, the LLM first attempted to extract an explicit date near the mention within the clinical note. If the date was parsed and passed validation checks, that timestamp was used, otherwise the note encounter date was assigned. Dates outside the study period or impossible values were rejected.

In generating patient timelines, to represent repeated instances of a data element abstracted from the complete selection of patient notes, we select the first valid timestamp per label class and display subsequent occurrences in gray.

## Results

### Reference datasets for performance evaluation

The test dataset of 125 notes (median length of 977 words, IQR 576 − 289), representing 125 unique patients with MM, was annotated for the selected data fields (100 notes annotated for all data fields, 25 additional notes for first-line therapy related data fields: *FLT regimen*,* FLT response*,* FLT response date*. Very few notes (< 10%) contained information for *FLT response date*; this data field was excluded from further analysis. Table 2 describes the patient cohort.

For the 12 data fields analyzed, the average annotators’ agreement measured by Krippendorff’s α was α = 0.77 for test and development set (0.83 for test set only), while average IRR-F_1_ was F_1_ = 0.74 for test and development set (0.79 for test set only). Values by data field are shown in Supplementary Table 2.

**Table 2:** Characteristics of overall, development and test set populations

Patient characteristics	Overall	Development set	Test set
No. of patients	250	125	125
Sex – male, n (%)	156 (62.4%)	77 (61.6%)	79 (63.2%)
Age at MM diagnosis (mean ± SD)	65.7 ± 10.6	67.4 ± 9.7	64.0 ± 11.2
**Race**			
White, n (%)	213 (85.2%)	106 (84.8%)	107 (85.6)
Black or African American, n (%)	21 (8.4%)	< 11	13 (10.4%)
Other*, n (%)	< 11	< 11	< 11
Unknown**, n (%)	< 11	< 11	< 11
**Ethnicity**			
Hispanic or Latino, n (%)	231 (92.4%)	116 (92.8%)	115 (92%)
Not Hispanic or Latino, n	< 11	< 11	< 11
Unknown, n	< 11	< 11	< 11
**Year of MM diagnosis**			
2019	58 (23.2%)	32 (25.6%)	26 (20.8%)
2020	58 (23.2%)	24 (19.2%)	34 (27.2%)
2021	54 (21.6%)	24 (19.2%)	30 (24.0%)
2022	55 (22%)	28 (22.4%)	27 (21.6%)
2023	25 (10%)	17 (13.6%)	< 11

*Asian, Native American and Pacific Islander, Indian Subcontinent

**Undisclosed, Unable to Provide

### Comparison of workflow performance

**Fig. 1:**
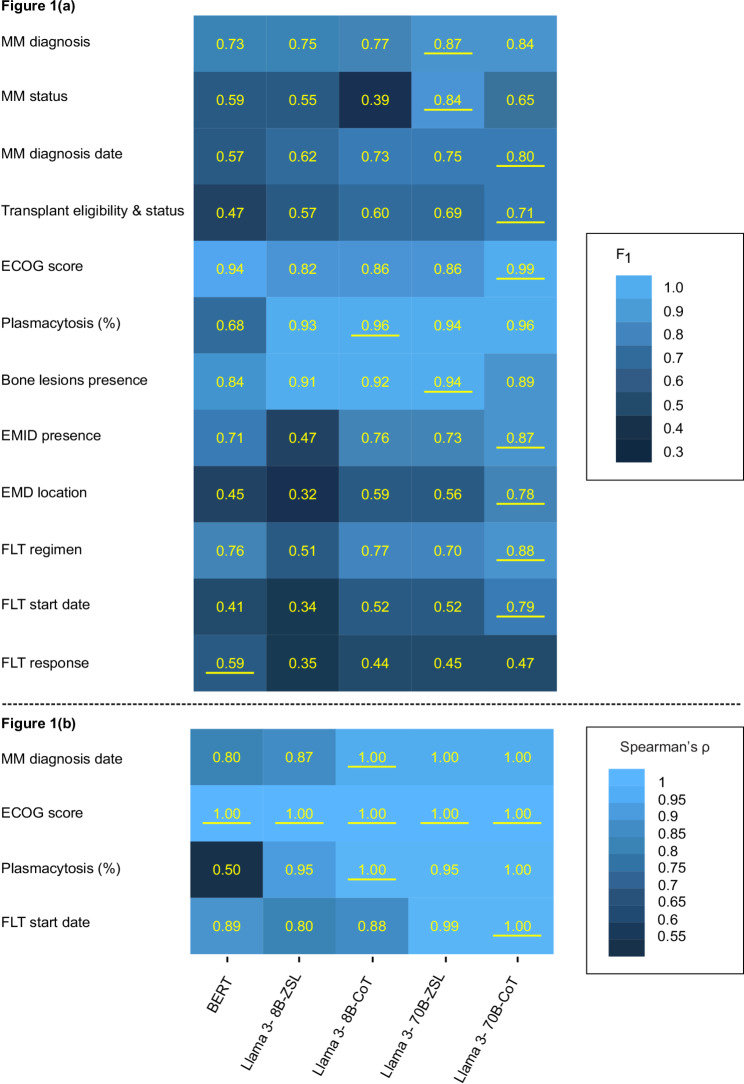
(**a**): F_1_ score by workflow and data field; (**b**): Spearman’s ρ for numeric data fields

In total, five LLM workflows (four Llama-based, and one BERT-based) were deployed. Performance of each workflow is shown in Fig. 1a. Spearman’s rank correlation coefficients were calculated for continuous data fields (*ECOG score*,* Plasmacytosis percentage*,* MM diagnosis date*, and *FLT start date*) (see Fig. 1b). Sensitivity analysis for the best-performing Llama workflow by data field is available in Supplementary Note 5. Performance for the date concepts (MM diagnosis date and FLT start date) across 7-, 30-, 60-, 90-, and 180-day buffers for each workflow is provided in Supplementary Note 5 (Supplementary Table 5a).

F_1_ statistics ranged widely by workflow and data field (F_1_ = 0.32–0.99). Llama 70B-CoT had the highest performance on seven out of 12 data fields overall. Notably, the BERT workflow outperformed Llama 8B with both prompt types for three fields, and outperformed 8B-ZSL in four additional fields. CoT generally boosted model performance, although three data fields (*Bone lesion presence*,* MM diagnosis*,* and MM status*) experienced worse performance with CoT than with ZSL in one or both Llama models. For data fields with continuous values, Spearman rank correlations of predictions with the reference data showed mostly near-perfect prediction by 70B workflows.

**Fig. 2:**
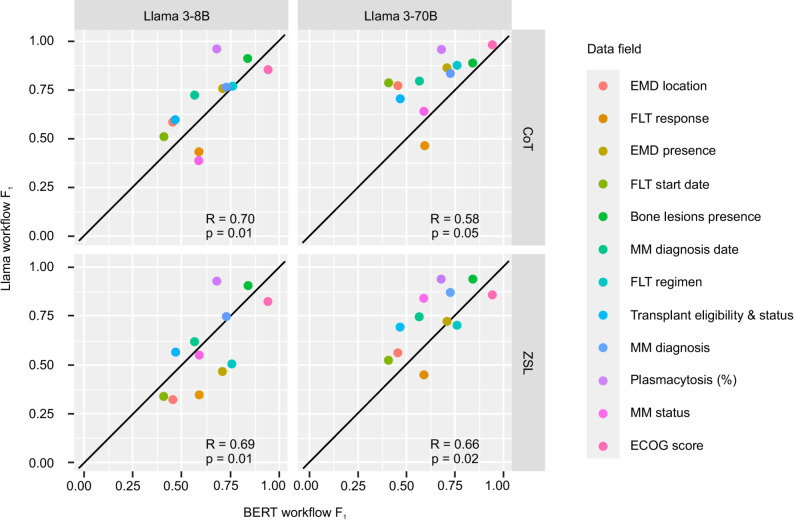
Llama vs. BERT workflow F_1_ by data field. R: Pearson correlation coefficient, p: p-value

Figure 2 demonstrates the F_1_ statistic distribution for each Llama workflow compared to BERT workflow. Larger model size and CoT prompting improve performance compared to BERT workflow more consistently, demonstrated by the distribution of data points above the equivalence line.

### Drivers of Llama workflow performance

ANOVA and pairwise analysis (see Tables 3 and 4) were used to quantify the impact of model size, prompting technique, and data field characteristics (type and IRR-F_1_) on Llama workflow F_1_ statistics. All variables significantly impacted Llama workflow performance (ANOVA *p* < 0.05). IRR-F_1_, capturing degree of abstraction difficulty for each data field, had the largest impact on workflow F_1_. Increasing model size (from Llama-8B to Llama-70B) had twice the impact on improving F_1_ as using CoT prompting instead of ZSL.

**Table 3:** ANOVA test for predictors of Llama workflow F_1_

Variable	df	MS	Statistic	η^2^	*p*-value	Significance
Model size (70B vs. 8B)	1	0.19	11.78	0.12	1.36 × 10^− 3^	**
Prompt style (CoT vs. ZSL)	1	0.08	4.97	0.05	3.12 × 10^− 2^	*
IRR-F_1_	1	0.36	21.70	0.21	3.20 × 10^− 5^	***
Data field type	2	0.18	10.92	0.21	1.52 × 10^− 4^	***
Residuals	42	0.02	NA	0.41	NA	

Legend: df – degrees of freedom, MS – mean squared, η^2^ – effect size

**Table 4a:** Univariate post-hoc testing of predictors of Llama workflow F_1_

Variable	Estimate	Statistic	*p*-value	Significance	Test
Model size (70B vs. 8B)	0.13	6.79	6.37 × 10^− 7^	***	Paired t-test
Prompt style (CoT vs. ZSL)	0.08	3.14	4.57 × 10^− 3^	**	Paired t-test
IRR-F_1_	1.02	3.52	9.87 × 10^− 4^	**	OLS
Data field type	*(See pairwise below)*	5.09	1.01 × 10^− 2^	*	ANOVA

**Table 4b:** Pairwise Tukey HSD post-hoc comparison of data field type on Llama workflow F_1_

Data field type	Estimate	Statistic	*p*-value	Significance	Test
Binary - categorical	0.18	2.57	0.01	*	Paired t-test
Binary - numeric	0.04	0.48	0.64		Paired t-test
Categorical - numeric	−0.15	−2.61	0.01	*	Paired t-test

### Comparing NLP workflow performance by inter-rater reliability score

**Fig. 3:**
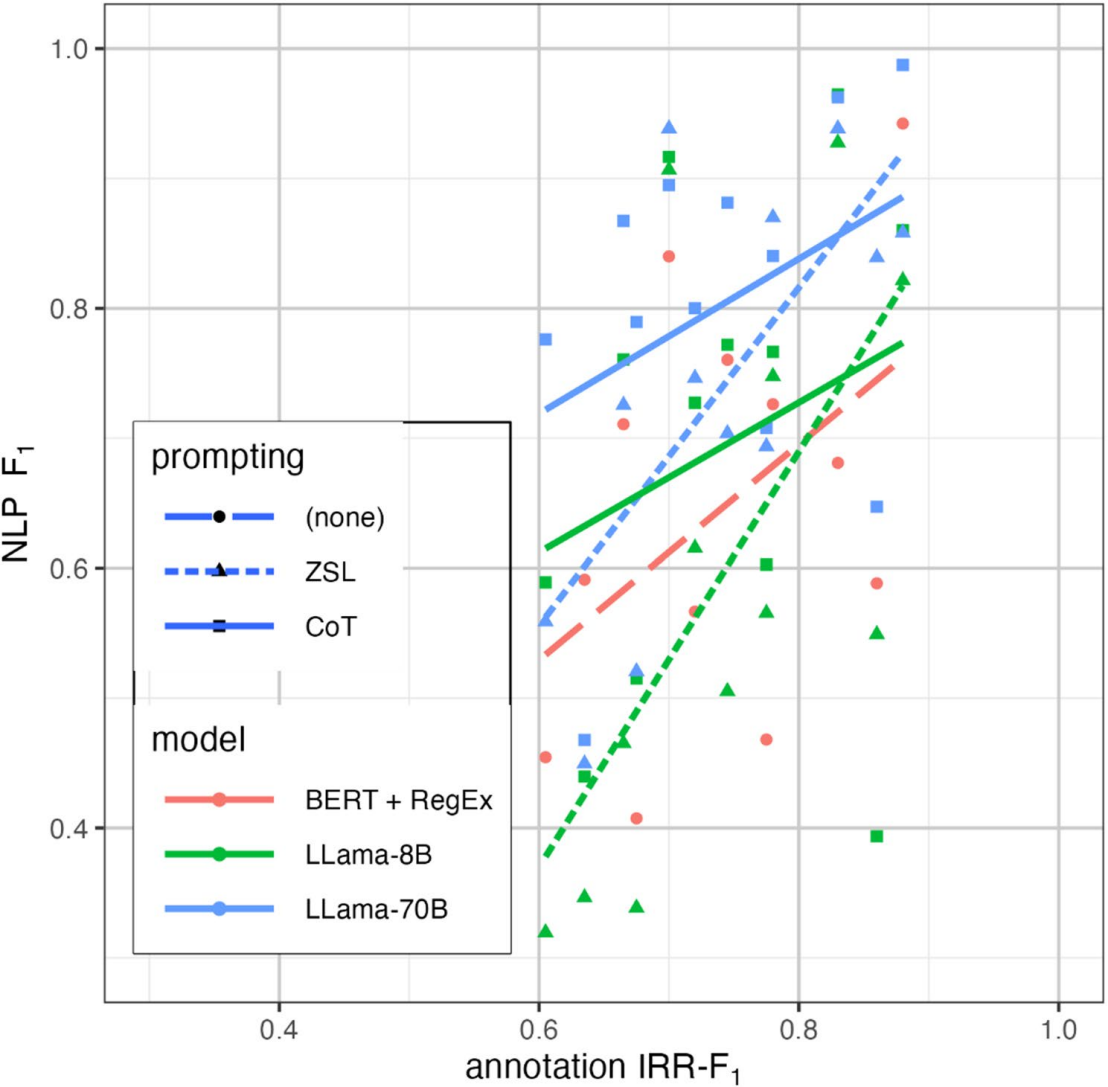
Trends in model performance (measured by F_1_) versus difficulty of abstraction (measured by IRR-F_1_), by model

As shown in Fig. 3, IRR-F_1_ varied across data fields from 0.61 to 0.88. For all data fields, the larger 70B model consistently dominated LLM workflow performance, with trendlines above the 8B and BERT model. At an IRR-F_1_ of approximately 0.8–0.85, the trendlines for Llama 8B intersect the BERT trendline, and Llama ZSL trendlines intersect CoT trendlines. The steeper slope of the trendlines for ZSL versus CoT reflect the heterogeneous response to prompting techniques, with CoT prompting boosting performance in the more difficult data fields but reducing performance in the fields with higher IRR-F_1_.

### Exploratory analysis of LLM-extracted events timing vs. structured diagnosis date

**Fig. 4:**
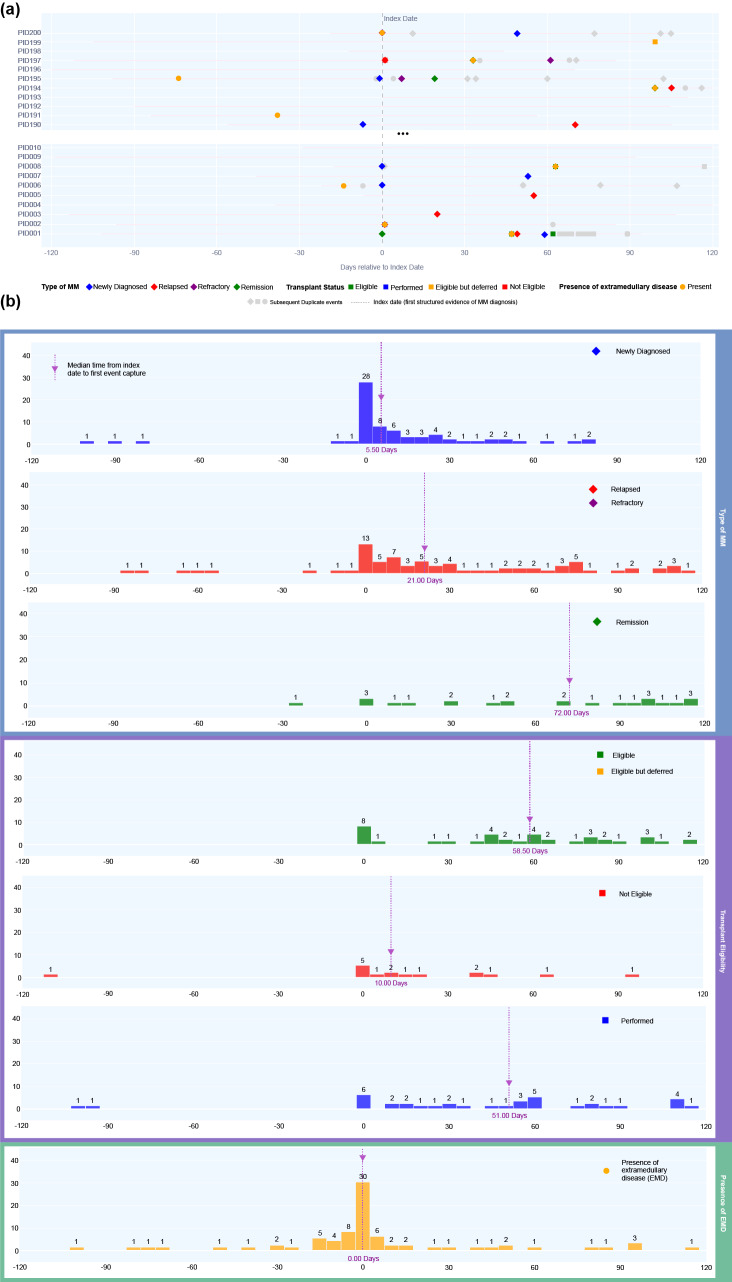
(**a**) Illustrative synthetic patient-level timelines, synthesized using the event rates observed in the 200-patient sample, show extracted labels categorized by type of MM (diamonds), transplant eligibility (squares), and EMD presence (circles), plotted by days relative to the index date (vertical dashed line). First occurrences are colored, and subsequent duplicates are grey. (**b**) Histograms summarize the timing of first label occurrence, highlighting median extraction times and clustering patterns around the index date

In a 200-patient subcohort, data fields for *MM status*, *Transplant eligibility & status*, and *EMD presence* were extracted with their associated time-stamps from all notes within +/-120 days of MM diagnosis date (by diagnosis code) by the top-performing LLM and plotted on a timeline. Figure 4a shows patient-level timelines for 20 illustrative patients (to protect patient-level data), demonstrating the recurrence of labels in the window around the MM diagnosis date. Figure 4b shows the aggregated distribution of first label occurrence in each data field for the entire 200-patient cohort. The most frequent labels occur within a narrow window around the diagnosis date. “Newly diagnosed” and “EMD present” labels cluster most closely to the structured diagnosis date, versus other labels which show more dispersed incidence. Review by authors with medical training (ASB, AOD, AK, MM, SKR, GV) concluded that these distributions were clinically reasonable based on timing of diagnostic testing, disease progression and follow-up, and documentation.

## Discussion

To accelerate RWD generation, time and resource efficiency in information extraction from unstructured text is paramount. LLMs are increasingly used to improve clinical IE efficiency but must be deployed judiciously. We tested five NLP workflows for clinical IE to understand performance variability and infer appropriate selection of NLP technique, model size, and prompt design.

Our workflows show performance variability across 12 data fields (F_1_ = 0.59—0.99 for the best NLP workflow). Subsequent statistical analysis of Llama workflows indicated that inter-rater reliability, model size, and prompt design were all significantly associated with performance (ANOVA *p* < 0.05). Inter-rater reliability (measured by IRR-F_1_) was associated with the impact of the largest magnitude on NLP workflow performance (measured by F_1_); a point increase in IRR-F_1_ was associated with a point increase in NLP workflow F_1_.

Given the statistically significant association and equivalent magnitude of impact, we suggest IRR-F_1_ as a novel guide for selecting an IE approach. All abstraction techniques, both human and machine, are imperfect. Even rigorously trained human abstractors err at a rate of 1–2%; to correct for this, double abstraction with expert arbitration is the gold standard [49]. Our analysis demonstrates that human error rate, quantified as IRR-F_1_, can be used a surrogate for degree of abstraction difficulty when analyzing Llama workflow performance. Since reference dataset creation is a standard NLP workflow development step, IRR-F_1_ can be calculated readily.

There is limited literature describing the necessary quality of NLP-extracted data versus expert abstracted data. In general, there is no accepted performance threshold for NLP-extracted RWD; assessment of model performance is a fit-for-purpose exercise. Hernandez-Boussard et al. (2019) propose a theoretical threshold of 85% recall and 90% sensitivity (which equates to an F1 score of approximately 87%) [50]. Empirical studies have shown that machine-learning generated RWD with an F_1_ score of ~ 85% generates comparable cohorts and similar findings on key outcomes to manually abstracted cohorts, although this F_1_ score was not shown to be a minimum [51, 52]. Other research shows that a prognostic model trained on NLP-extracted RWD with a low performance (F_1_ ~ 0.5) can still achieve a high AUROC (~ 0.8) [53]. 

Our data shows that IRR-F_1_ of 0.8 is a reasonable threshold for flagging data field difficulty, below which larger models are required to reach adequate data quality and where CoT prompting improves model results. Llama 70B is recommended when available and unconstrained by resources. CoT prompting is required for more difficult data fields, while ZSL prompting may be sufficient or even superior when IRR-F_1_ is > 0.8. Under resource constraints, BERT and Llama 8B models are largely adequate for IRR-F_1_ > 0.8. For difficult fields with IRR-F_1_ < 0.8, researchers without access to a larger model comparable to Llama 70B will need to consider more labor-intensive options, such as manual abstraction for challenging fields or potentially fine-tuning small models.

**Fig. 5:**
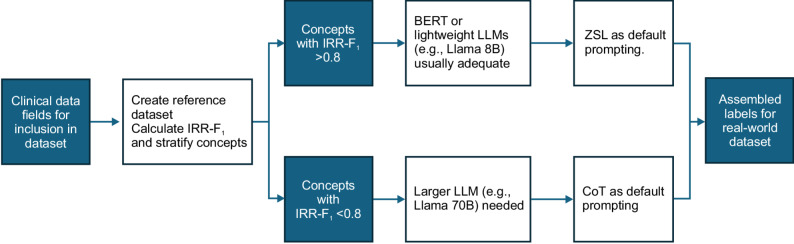
Evaluation criteria for designing IE workflow by data field

Figure 5 summarizes our recommendation for prioritizing resources for data field extraction.

Finally, we constructed timelines of data field mentions over the complete patient record for select clinical concepts, allowing review for clinical reasonableness and revealing label redundancy in patient charts. Clinical documentation is inherently incomplete, and clinical characteristics evolve along the clinical journey, e.g., a radiology report may not mention transplant status; disease status may change from newly diagnosed to relapsed. NLP tool optimization will not surmount information missingness from a single note or changes over time. Patient journey timelines with repetition of data labels could help solve the uncertainty or incompleteness of any single note when generating labels for a real-world data set. Deploying NLP models over a patient record, rather than a single note, and deriving a consensus label within a timeframe could produce more accurate labelling. Development of a logical method to using information repetition demonstrated along the patient journey to create higher-accuracy real-world datasets would extend this research.

This analysis has two limitations inherent to LLM utilization. LLM performance is highly sensitive to prompt design, which is difficult to quantify, and to model pre-training, which here is limited to the corpora used for Llama models. We could not exhaustively test all models or potential prompts; demonstrated applications of this proposed approach using different NLP models and prompts will support generalization.

## Conclusion

This study analyzes the performance of various NLP models (BERT-based and foundation open-source LLMs of different sizes) in extracting data fields relevant to MM for RWD generation. We show that inter-rater reliability is a statistically significant predictor of meaningful magnitude for NLP performance. We propose using an inter-rater reliability metric as a novel guide to efficiently approach information extraction for real-world dataset creation for time and resource allocation without sacrificing data quality.

## Supplementary Information

Below is the link to the electronic supplementary material.

Supplementary Material 1: Supplementary Note 1: Data selection and evaluation metrics

Supplementary Material 2: Supplementary Note 2: Abstraction protocol and metrics

Supplementary Material 3: Supplementary Note 3: Llama workflow prompts

Supplementary Material 4: Supplementary Note 4: BERT workflow rules and model performance metrics

Supplementary Material 5: Supplementary Note 5: Llama workflow sensitivity analysis

## Data Availability

This study involves the analysis of de-identified Electronic Health Record (EHR) data via the nference Analytics Platform. The data shown and reported in this manuscript were extracted from this environment using an established protocol for data extraction, aimed at preserving patient privacy. The data has been de-identified pursuant to an expert determination in accordance with the HIPAA Privacy Rule. Any data beyond what is reported in the manuscript, including but not limited to the raw EHR data, cannot be shared or released due to the parameters of the expert determination to maintain data de-identification. For additional details regarding the nference Analytics Platform, please contact the corresponding authors.

## Code availability

Annotation protocol, Llama workflow prompts, and BERT workflow rules and model performance metrics are available in Supplementary Materials.

## Abbreviations

AMC: Academic medical center
BERT: Bidirectional Encoder Representations from Transformers
COT: Chain-of-thought
CPT: Current Procedural Terminology
EHR: Electronic Health Record
EMD: Extra-medullary disease
FLT: First line therapy
HCP: Healthcare provider
HCPCS: Healthcare Common Procedure Coding System
IE: Information extraction
IMWG: International Myeloma Working Group
IRR: Inter-rater reliability
IRR-F_1_: Inter-rater reliability as measured by the agreement between each abstractor and the final arbitrated label evaluated using an average F_1_ score between each annotator’s and the consensus labels
K-α: Krippendorff’sα
LLM: Large language models
MM: Multiple myeloma
NDC: National Drug Code
NER: Named entity recognition
NLP: Natural language processing
RWD: Real world data
RWE: Real world evidence
ZSL: Zero-shot-learning
