# Supplementary material for: Automated abstraction of clinical parameters of multiple myeloma from real-world clinical notes using large language models
Source: BMC Med Inform Decis Mak. 2026 Jan 28;26:51. doi: 10.1186/s12911-026-03345-z (PMC12924526; doi:10.1186/s12911-026-03345-z)
Supplement: Supplementary file 1 — Supplementary Material 1: Supplementary Note 1: Data selection and evaluation metrics [file 12911_2026_3345_MOESM1_ESM.pdf]

Supplementary Note 1: Data Selection & Evaluation Metrics

1.1 Data selection

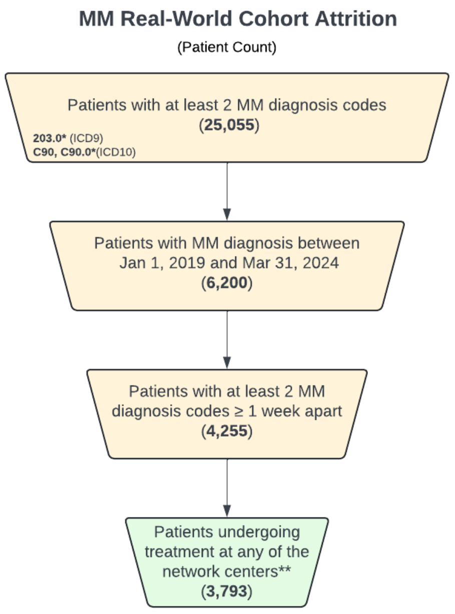

Supplementary Figure 1.1a. Cohort attrition diagram: Structured codes 203.0*(ICD-9), and C90, C90.0* (ICD-10) were used for multiple myeloma; * indicates all the children codes within the parent code. ** criteria for determining treatment were assumed if patient had at least one hospital encounter within six months of the diagnosis date and another beyond six months.

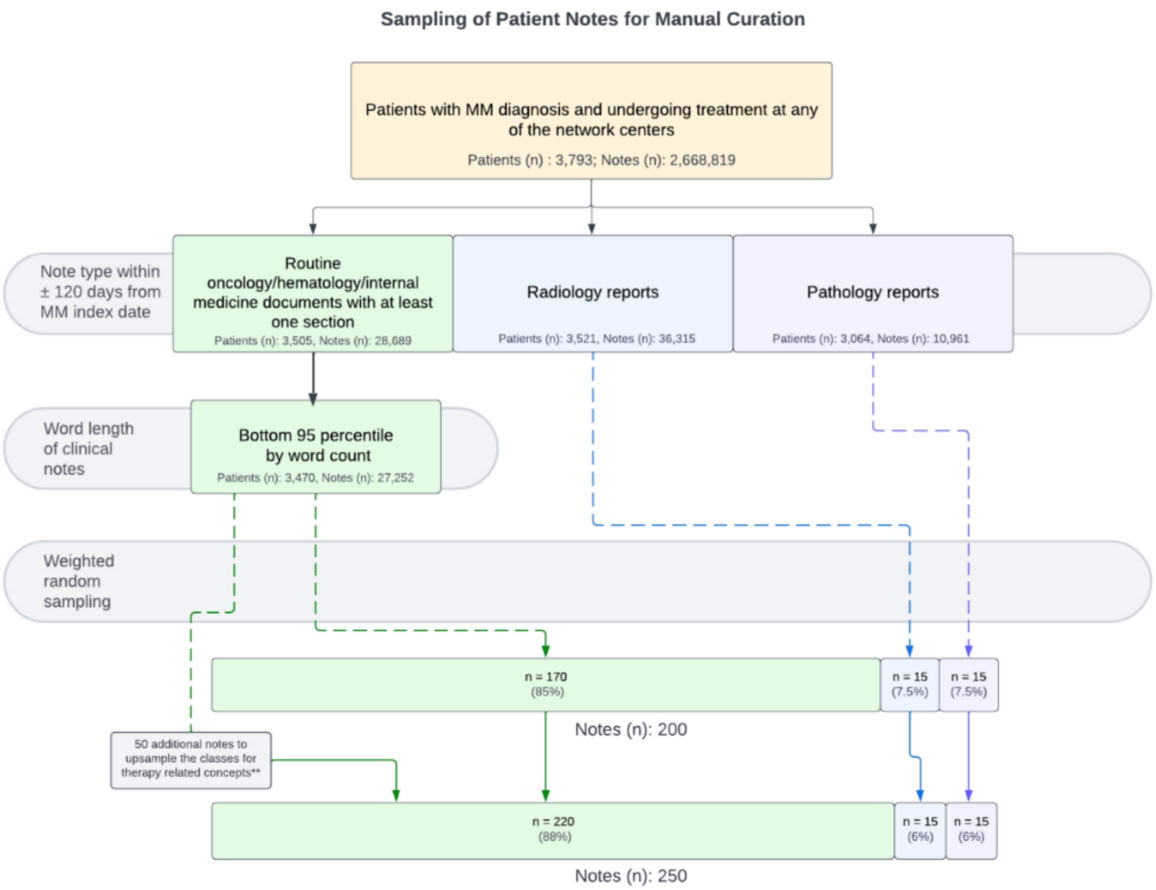

Supplementary Figure 1.1b. Sampling and note extraction pipeline:
***Therapy-related data fields include first-line therapy (FLT), date of start of FLT, response to FLT and date of recorded response to FLT.*

Notes within 120 days of diagnosis date and either (1) routine clinical notes from internal medicine, oncology and hematology services; (2) radiology reports; and (3) pathology reports were retained. The longest 5% were excluded because they challenged the optimal context length of the LLMs. On review, the main driver of high word count was duplicative text, likely due to copy-pasting from prior notes. 200 notes were selected using weighted random sampling proportional to frequency of note type.

- 85% notes from internal medicine, oncology, or hematology practice
  - 40% sampled on diagnosis date
  - 40% furthest note within ±120 days of the diagnosis date
  - 20% elsewhere within ±120 days of the diagnosis date
- 7.5% from Radiology practice within ±120 days of the diagnosis date
- 7.5% from Pathology practice within ±120 days of the diagnosis date

50 additional notes were selected because therapy-related data fields (first-line therapy (FLT), date of start of FLT, response to FLT and date of recorded response to FLT) had low coverage after abstraction. The total sampled notes represent 250 unique patients and were divided into development and test sets of 125 notes each.

1.2 Evaluation Metrics

Macro-F_1 scores were used to evaluate the performance of the NLP workflows. For multi-label fields such as dates, macro-F_1 score was substituted with a weighted (task-specific) F_1. Data fields with continuous values were assessed using Spearman's rank correlation coefficient (Spearman's ρ).

Supplementary Table 1.2a: Weighted (task-specific) F_1 metric definitions

**Condition**	**Ground Truth (GT)**	**Prediction**	**Classification Outcome**
Ground truth and prediction both present and match	Captured	Captured and matches ground truth	True Positive (TP)
Ground truth and prediction both absent	Not captured	Not captured	True Negative (TN)
Ground truth absent but prediction present	Not captured	Captured	False Positive (FP)
Ground truth present but prediction absent	Captured	Not captured	False Negative (FN)
Ground truth present and prediction present, not matched	Captured	Captured but does not match	False Positive (FP)

Supplementary Table 1.2b: Data fields and evaluation metric used.

Concept	Data field	Value type and range	Evaluation metric
MM disease state	*1. MM diagnosis*	Categorical<br>*Yes / Likely / No / Unspecified**	Macro F_1
	2. MM status	Categorical<br>*Newly diagnosed / Relapsed / Refractory / Remission*	Macro F_1
	3. MM diagnosis date	Datetime<br>*YYYY-MM-DD*	Task-specific F_1 score, Spearman's ρ
Transplant eligibility & status	*4. Transplant eligibility & status*	Categorical<br>*Eligible / Eligible but deferred / Ineligible / Performed / Unspecified**	Macro F_1
Eastern Cooperative Oncology Group (ECOG) performance status scale	*5. ECOG score*	Integer<br>*0—5*	Macro F_1, Spearman's ρ
Plasmacytosis	*6. Plasmacytosis percentage*	Integer<br>*0-100%*	Macro F_1, Spearman's ρ
MM-related bone lesions	*7. MM-related bone lesion presence*	Binary<br>*Yes / No / Unrelated / Unspecified**	Macro F_1
Extra-medullary disease (EMD)	*8. EMD presence*	Binary<br>*Present / No / Unspecified**	Macro F_1
	9. EMD location	Binary<br>*Paramedullary / Soft tissue / Unspecified**	Macro F_1
HCP-documented First line therapy (FLT)	*10. FLT regimen*	Categorical<br>*Set of drugs and procedures, mapped to NDC or RxNorm and HCPCS or CPT***	Task-specific F_1 score
	11. FLT start date	Datetime<br>*YYYY-MM-DD*	Task-specific F_1 score, Spearman's ρ
HCP-documented FLT response	*12. FLT response*	Categorical<br>*IMWG 2016 response categories: Stringent Complete Response / Complete Response / Very Good Partial Response / Partial Response / Minimal Response /<br>No Response / Progressive Disease / Unspecified**	Macro F_1
	13. FLT response date	Datetime<br>*Formatted as YYYY-MM-DD*	Task-specific F_1 score, Spearman's ρ
