# Supplementary material for: Automated abstraction of clinical parameters of multiple myeloma from real-world clinical notes using large language models
Source: BMC Med Inform Decis Mak. 2026 Jan 28;26:51. doi: 10.1186/s12911-026-03345-z (PMC12924526; doi:10.1186/s12911-026-03345-z)
Supplement: Supplementary file 5 — Supplementary Material 5: Supplementary Note 5: Llama workflow sensitivity analysis [file 12911_2026_3345_MOESM5_ESM.pdf]

Supplementary Note 5 - LLM Sensitivity Analysis

Temperature and top-k hyperparameter settings were varied to explore the robustness of the prompts and prompting techniques used for extraction.

For hyperparameter sensitivity analyses, the best-performing Llama workflows were run with 6 pairs of temperature (values of 10, 50) and top-k (values of 0.4, 0.7 and 1.0) values. For each pair, the experiment was repeated 10 times with different seeds. The resulting F1-scores from these 10 runs were averaged to estimate the variance in accuracy and to assess the overall robustness of the LLM workflows used for each clinical concept.

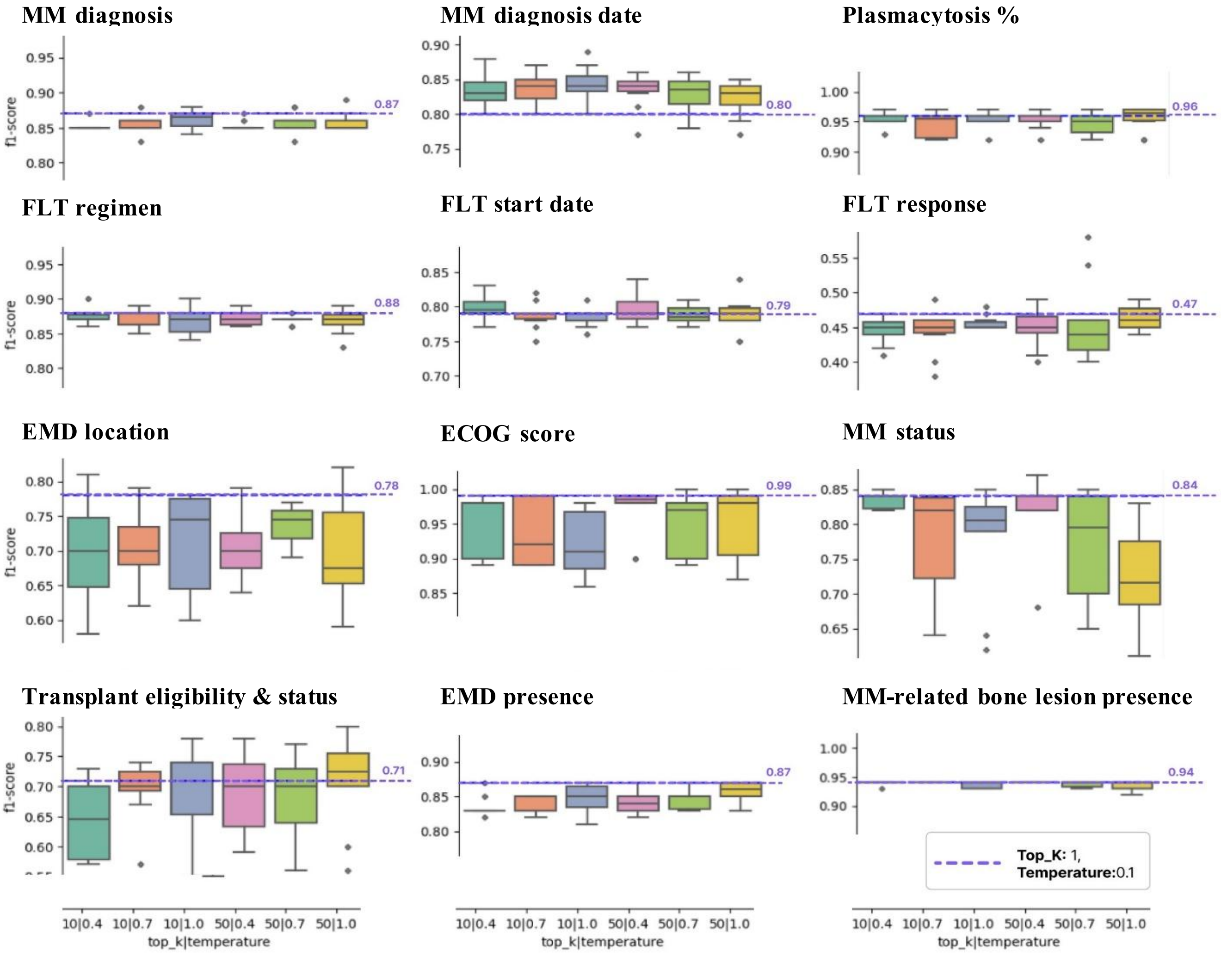

Supplementary Figure 5: Performance variance over 10 iterations with random seeds for different combinations of temperature and top k values for the best-performing LLM workflow in the test set. The purple dotted line indicates the performance with top-k=1 and temperature=0.1

Supplementary Table 5a. Performance metrics of date concepts with a buffer threshold						
**Concept**	**Buffer days**	**F_1 score**				
		**BERT**	**Llama 3-8B-ZSL**	**Llama 3-8B-CoT**	**Llama 3-70B-ZSL**	**Llama 3-70B-CoT**
MM diagnosis date	7	0.6	0.67	0.74	0.85	0.8
	30	0.65	0.74	0.77	0.86	0.83
	60	0.69	0.76	0.83	0.88	0.86
	90	0.71	0.79	0.83	0.91	0.88
	180	0.71	0.8	0.83	0.91	0.88
FLT start date	7	0.44	0.39	0.58	0.54	0.81
	30	0.49	0.41	0.66	0.6	0.84
	60	0.49	0.43	0.66	0.62	0.85
	90	0.49	0.43	0.66	0.65	0.85
	180	0.52	0.43	0.66	0.68	0.85

Supplementary Table 5a. Performance of five LLM workflows (four Llama-based, and one BERT-based) in the date concepts (MM diagnosis date and FLT start date) across 7-, 30-, 60-, 90-, and 180-day buffers for each workflow.
